# Operando direct observation of spin-states and charge-trappings of blue light-emitting-diode materials in thin-film devices

**DOI:** 10.1038/s41598-020-75668-4

**Published:** 2020-11-02

**Authors:** Fumiya Osawa, Kazuhiro Marumoto

**Affiliations:** 1 Division of Materials Science, University of Tsukuba, Tsukuba, Ibaraki 305-8573 Japan; 2 Tsukuba Research Center for Energy Materials Science (TREMS), University of Tsukuba, Tsukuba, Ibaraki 305-8570 Japan

**Keywords:** Materials for devices, Applied physics

## Abstract

Spin-states and charge-trappings in blue organic light-emitting diodes (OLEDs) are important issues for developing high-device-performance application such as full-color displays and white illumination. However, they have not yet been completely clarified because of the lack of a study from a microscopic viewpoint. Here, we report operando electron spin resonance (ESR) spectroscopy to investigate the spin-states and charge-trappings in organic semiconductor materials used for blue OLEDs such as a blue light-emitting material 1-bis(2-naphthyl)anthracene (ADN) using metal–insulator–semiconductor (MIS) diodes, hole or electron only devices, and blue OLEDs from the microscopic viewpoint. We have clarified spin-states of electrically accumulated holes and electrons and their charge-trappings in the MIS diodes at the molecular level by directly observing their electrically-induced ESR signals; the spin-states are well reproduced by density functional theory. In contrast to a green light-emitting material, the ADN radical anions largely accumulate in the film, which will cause the large degradation of the molecule and devices. The result will give deeper understanding of blue OLEDs and be useful for developing high-performance and durable devices.

## Introduction

Organic light-emitting diodes (OLEDs) have features such as highly efficient spontaneous-light-emission and flexibility and have attracted much attention as higher-generation displays and lighting^1–4^. A practical fluorescent OLED has been reported in 1987^5^, then phosphorescent OLEDs have been developed^6^. More recently, efficient OLEDs utilizing thermally activated delayed fluorescence (TADF) have been reported, and they have been extensively studied from a fundamental viewpoint^7,8^. However, fluorescent materials have been still used mainly as luminescent materials for practical OLEDs from viewpoints of material cost, lifetime, and stability. Many studies have been performed on fluorescent OLEDs with a typical green light-emitting material tris(8-hydroxyquinoline) aluminum (Alq_3_)^5,9–13^. However, studies on blue light-emitting materials have not yet been fully performed with respect to high efficiency and long lifetime, compared to those on red or green light-emitting materials, because of the wide bandgap and the problem of complicated multilayered structures of blue OLEDs^14–25^. Such study on blue OLED materials in addition to red and green OLED materials is important to further develop products such as high-performance full-color display.

Blue OLED materials need the wide bandgap between energy levels of highest occupied molecular orbital (HOMO) and lowest unoccupied molecular orbital (LUMO). Owing to this reason, there have been problems for the device-performance improvement of blue OLEDs because of the difficulties such as stable molecular design, the increase in nonradiative deactivation rate by trapping sites, the increase in the difficulty in manufacturing high-efficiency devices by multilayered structures, etc^14–25^. It is not easy to measure what kind of changes occurring during device operation in multilayered thin-films. The internal states inside the devices cannot be directly obtained with macroscopic measurements such as current–voltage characteristics, and the information inside the devices cannot be also measured from the surface observation of devices. Thus, it is very important to clarify the internal states of devices during device operation from a microscopic viewpoint because these states are essential to determine the device performance and durability.

Electron spin resonance (ESR) spectroscopy is one of the useful measurement methods to study the microscopic states of devices. The ESR method can evaluate materials at the molecular level using unpaired electrons as a probe and can directly observe films and devices nondestructively. In particular, the ESR method has a feature of operando evaluation for the inside of devices. With this ESR method, studies have been performed from a microscopic viewpoint such as the spin-states of charges in organic devices, the charge-trappings in thin-films and at interfaces, and the orientation of molecules^26–34^. For OLED research, the correlation between the formation of radical species and the luminance degradation during device operation has been discussed using an electron transport material 4,7-diphenyl-1,10-phenanthroline (BPhen)^21^ and the green fluorescent material Alq_3_^32^. However, no ESR research has been performed on the spin-states of blue OLED materials in addition to their devices during device operation.

Here, we report operando spin analysis of organic devices to directly elucidate the microscopic properties of blue OLED materials such as 1-bis(2-naphthyl)anthracene (ADN) in their devices. The reason for the use of ADN is to firstly study the blue OLED with a simple spin-state. That is, the fluorescent ADN is expected to show no complicated ESR signal due to triplet states that are expected for phosphorescent or TADF OLED materials. Metal–insulator–semiconductor (MIS) diode structures were fabricated, and the spin-states of the blue OLED materials and the charge dynamics in the devices have been clarified at the molecular level using operando ESR method, for the first time to the best of our knowledge. Clear ESR signals from the blue OLED materials were observed and their *g*-factors have been well reproduced by density functional theory (DFT). The existence of charge-trappings in the organic layers has been identified from the microscopic viewpoint. Moreover, the long-lived electron accumulation in ADN and Alq_3_ has been directly demonstrated at the molecular level. Electron or hole only devices and blue OLEDs have been studied with the ESR method for a comparison with the results of the MIS diodes. Our results will indicate that long-lived electron accumulation exists in blue OLEDs during device operation, and that such anion radicals react with oxygen and/or moisture in blue OLEDs during device operation, which will be one of the device-degradation causes, as discussed for Alq_3_-based OLEDs^9^.

## Materials and methods

**Figure 1:**
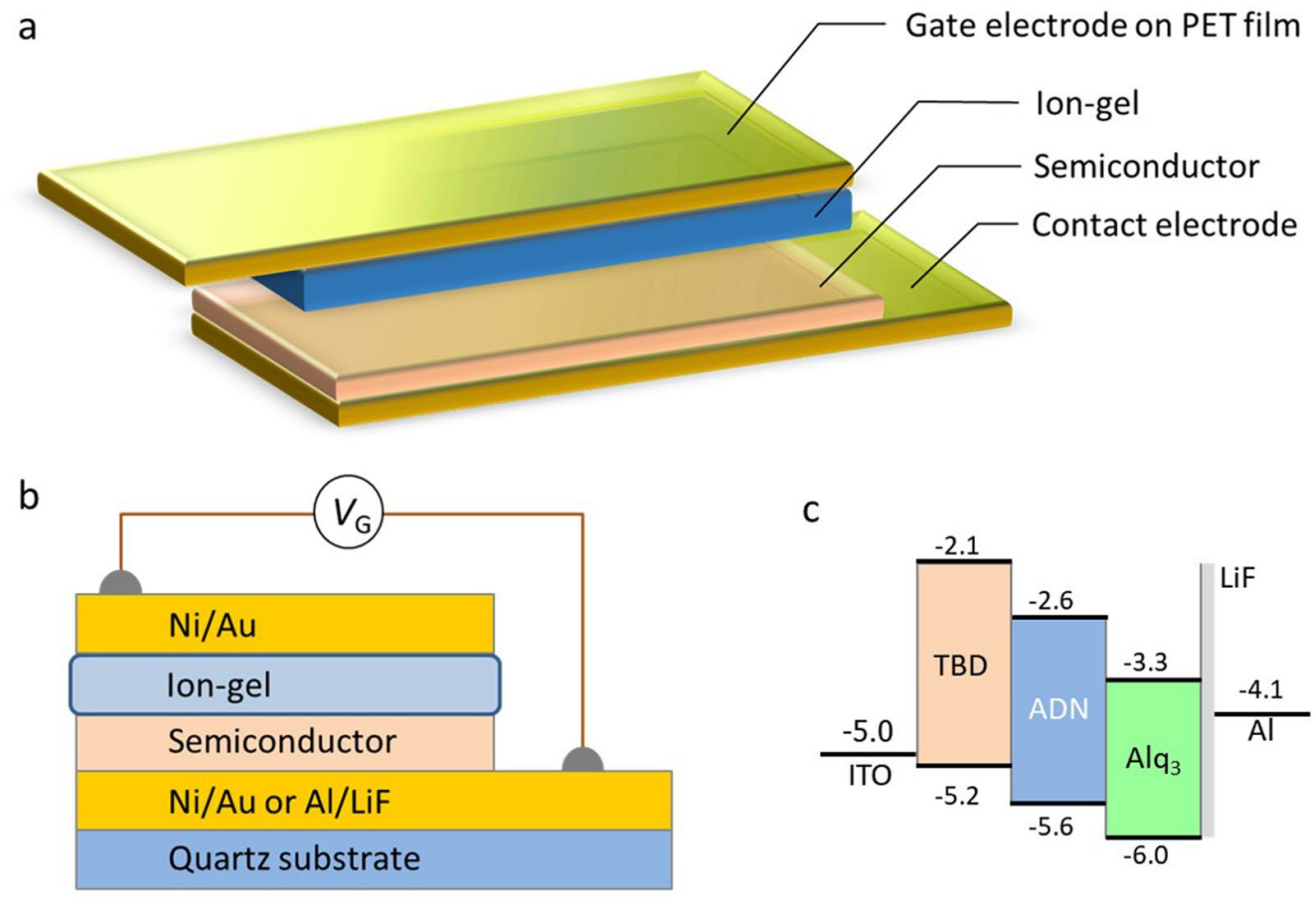
Schematic of an organic MIS diode and an energy diagram of a blue OLED. (**a**) Schematic structure of an organic semiconductor device used in this study. (**b**) Cross section of the device. (**c**) Energy diagram of the organic materials and electrodes of the blue OLED targeted in this study.

**Figure 2:**
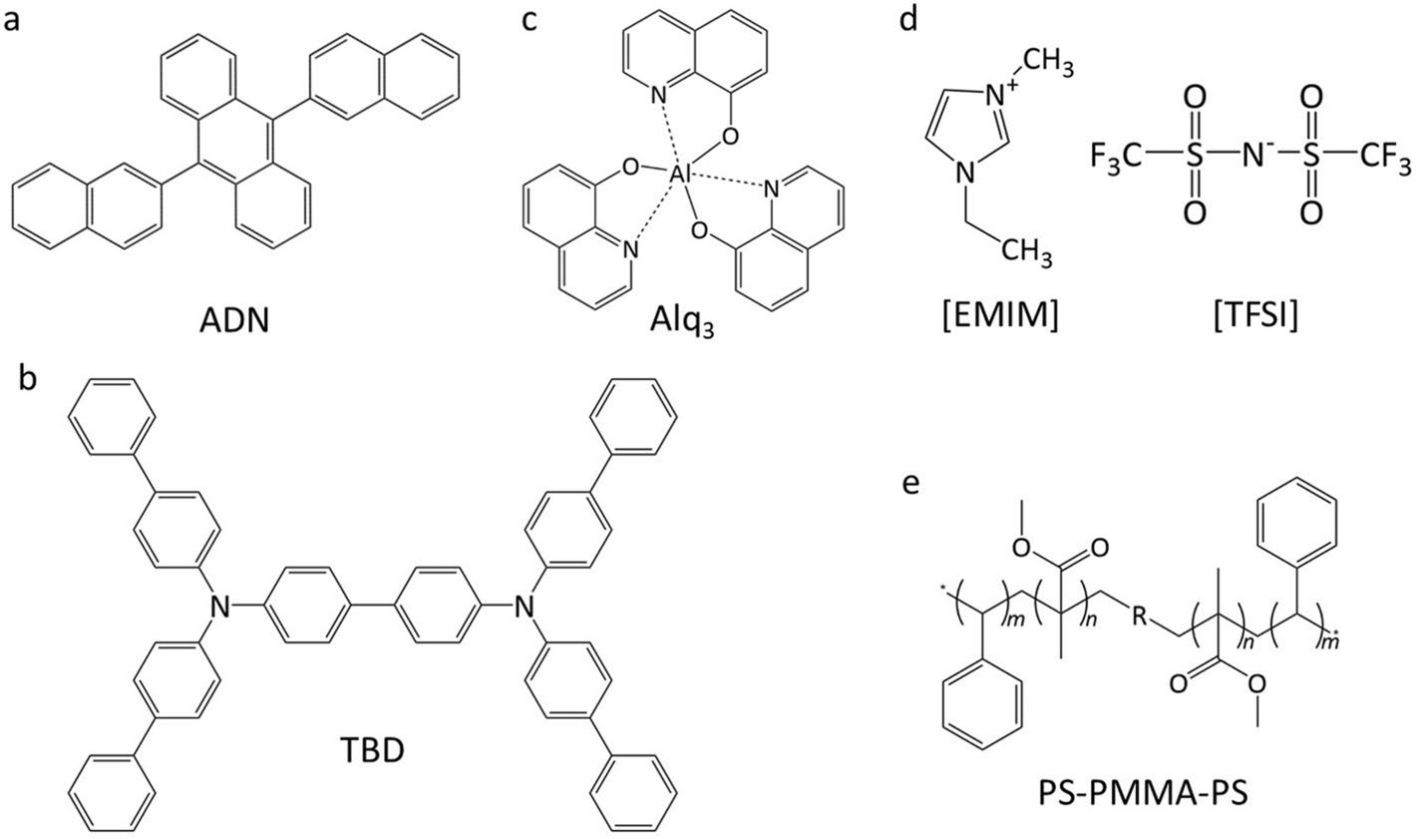
Chemical structures of the organic materials in the devices. (**a**,**b**,**c**) Chemical structures of organic semiconductor materials: (**a**) 9,10-bis(2-naphthyl)anthracene (ADN) as a blue light-emitting material, (**b**) *N*,*N*,*N’*,*N’*-tetrakis(4-biphenylyl)benzidine (TBD) as a hole transport material, and (**c**) tris(8-hydroxyquinoline) aluminum (Alq_3_) as an electron transport material. (**d**,**e**) Chemical structures of ion-gel materials: (**d**) ionic liquid 1-ethyl-3-methylimidazolium (EMIM) and bis(trifluoromethylsulfonyl)imide (TFSI), and (**e**) a gelator ABA-type triblock copolymer poly(styrene-*b*-methylmethacylate-*b*-styrene) (PS-PMMA-PS).

To attain a high signal-to-noise ratio of the ESR signal by increasing the device’s active area, we utilized a rectangular device structure (3 mm × 30 mm) in an ESR sample tube with an inner diameter of 3.5 mm^33,34^. The schematic of an organic MIS diode structure and the cross section are shown in Fig. 1a,b, respectively. The chemical structures of employed organic semiconductor materials are shown in Fig. 2. Figure 1c shows the energy diagram of the organic materials and electrodes of the blue OLED targeted in this study. A blue light-emitting material ADN, a hole transport material *N*,*N*,*N'*,*N'*-tetrakis(4-biphenylyl)benzidine (TBD), and an electron transport material Alq_3_ were used as semiconductor layers in the MIS diodes. An ion-gel was used as the insulator. High charge density in semiconductors and low-voltage device operation have been achieved with the high-capacitance ion-gel insulator forming electric double layers (EDLs) at the semiconductor/insulator interfaces^31,35–37^. The ion-gel is composed of ionic liquid 1-ethyl-3-methylimidazolium (EMIM) and bis(trifluoromethylsulfonyl)imide (TFSI), and a gelator ABA-type triblock copolymer poly(styrene-*b*-methylmethacylate-*b*-styrene) (PS-PMMA-PS) (Fig. 2). Ni/Au or Al/LiF contact electrode was used for hole or electron injection, respectively, which was fabricated on a quartz substrate with a vacuum-deposition method (Fig. 1b). Ni/Au gate electrode was deposited on a polyethylene terephthalate (PET) substrate with the vacuum-deposition method. Here, Ni was used as an adhesion layer between Au and quartz or PET substrate. The layers of ADN, TBD, and Alq_3_ were formed on the contact electrode with the vacuum-evaporation method. An ion-gel was deposited on the gate electrode with a drop-casting method. Then the ion-gel was placed on the semiconductor layer, completing the MIS diode. The fabricated device was sealed in the ESR sample tube together with a desiccant in a nitrogen-filled glove box to suppress the influence by oxygen and moisture^10^. For a companion with the studies of the MIS diodes, we have fabricated hole or electron only devices and the above-mentioned blue OLEDs (Fig. 1c) with a method similar to that mentioned above.

The fabricated MIS diodes, hole or electron only devices, and blue OLEDs were measured with an ESR spectrometer and a source meter. A standard Mn^2+^ marker sample was used to calibrate the *g*-factor, the ESR linewidth, and the number of spins. The *g*-factor was evaluated from the resonance magnetic field where the ESR spectrum with a first derivative form has a value of zero. The peak-to-peak ESR linewidth (Δ*H*_pp_) was evaluated as the difference between the two magnetic fields at a peak and valley in the ESR spectrum. The gate-voltage *V*_G_ was applied to the MIS diodes, and the ESR measurements were performed at room temperature. The direction of the external magnetic field *H* was parallel to the substrate plane unless otherwise stated. Further details of experimental methods are described in the “Methods” section.

The reasons why we use the MIS diodes are as follows. First, the use of the MIS diodes clearly selects the electric charge polarity such as cations and anions in the organic layer in the devices. This selection is important to identify the nature of charge species in the organic layer. In the case of OLEDs, it is usually hard to select the charge polarity in the organic layer. Second, we utilize a continuous-wave ESR method with a modulation frequency of 100 kHz for the *H*, that is, lock-in detection, in our ESR experiments^30–34^. Thus, the charge carriers with a lifetime of < 10 μs in OLEDs, which contribute to the standard OLED operation, cannot be observed using the present ESR method^30–34^. Here we mean the term of “lifetime” for the duration of the existence of charge carriers in devices. Thus, the observed ESR signals are due to charge carriers with a lifetime of > 10 μs in devices, namely, accumulated (or deeply trapped) carriers^30–34^. The lifetime of charges in the MIS diodes are enough longer than 10 μs because the charges in the semiconductor are accumulated in the MIS diodes. The 10-μs lifetime is calculated from one cycle of the *H* modulation frequency of 100 kHz, that is, 1/(100 × 10^3^) (1/s^−1^). In the case of OLEDs, a typical lifetime of charge carriers in devices is estimated to be < 10 μs when we use the following parameters according to the literatures^1–3^: a mobility of > 2 × 10^−6^ cm^2^ V^−1^ s^−1^, an electric field in a device of 5 × 10^5^ V cm^−1^, and a film thickness of 100 nm. This estimated lifetime is much shorter than that in the MIS diodes.

## Results and discussion

### Operando ESR spectra of organic devices

**Figure 3:**
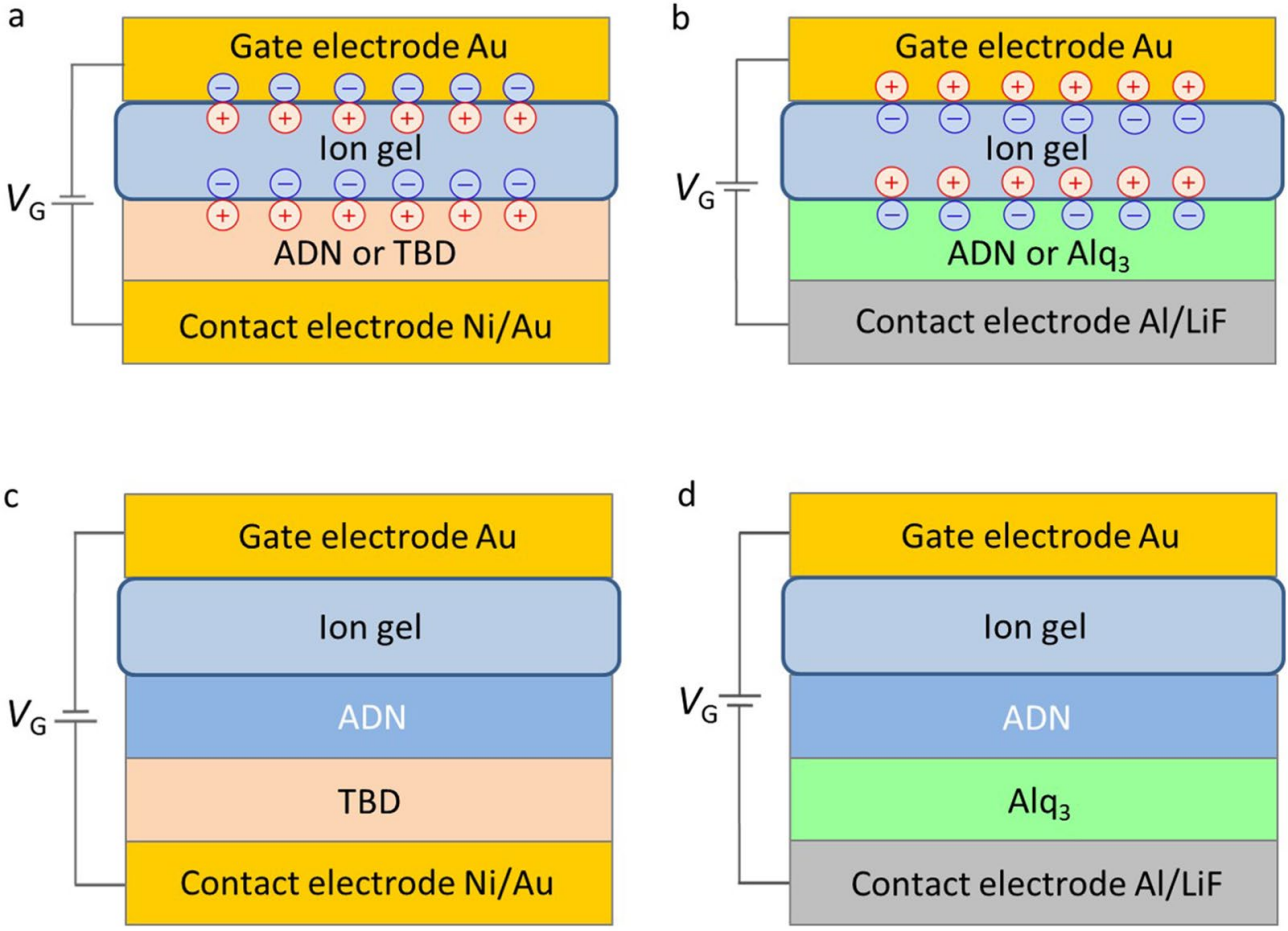
Schematics of the cross sections of organic devices. (**a**) MIS diode with ADN (60 nm) or TBD (100 nm) for hole accumulation at negative *V*_G_. (**b**) MIS diode with ADN (60 nm) or Alq_3_ (60 nm) for electron accumulation at positive *V*_G_. In (**a**) and (**b**), the formation of electric double layers is shown. (**c**) Organic multilayer MIS diode with TBD/ADN. (**d**) Organic multilayer MIS diode with ADN/Alq_3_.

First, we present the spin-states of the blue light-emitting material ADN, the hole-transport material TBD, and the electron-transport material Alq_3_ in the MIS diodes using the operando ESR spectroscopy. Figure 3a,b outline the charge-accumulation mechanisms. When the ion-gel is polarized by a negative (or positive) *V*_G_, holes (or electrons) are electrically accumulated in the semiconductor at the semiconductor/ion-gel interface (Fig. 3a,b). In this case, an EDL is formed at the interface, which increases the electric capacity and decreases the charge-injection threshold voltage; the capacitance increase is one order of magnitude higher than that of MIS diodes with conventional solid insulators^31,35–37^.

**Figure 4:**
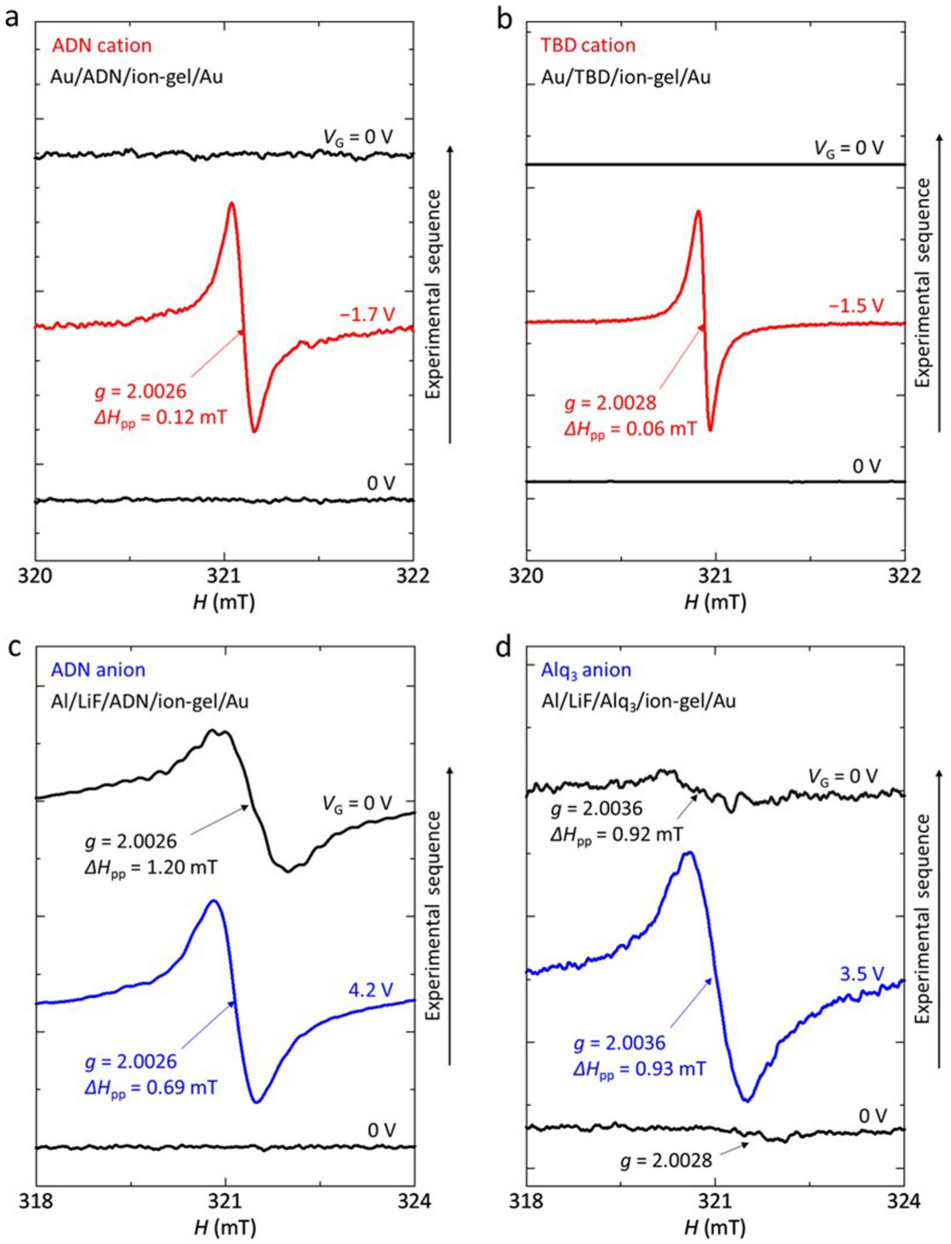
*V*_G_ dependence of the ESR spectra of the organic MIS diodes. (**a**,**b**) ESR spectra of the ADN device (**a**) and the TBD device (**b**) in negative *V*_G_ region. (**c**,**d**) ESR spectra of the ADN device (**c**) and the Alq_3_ device (**d**) in positive *V*_G_ region. These measurements were performed at the external magnetic field *H* parallel to the substrate plane at room temperature (RT).

The operando ESR spectra with the MIS diodes are shown in Fig. 4. In each figure, each data has the same scale in the axis of ordinate to compare the magnitude variation by *V*_G_, that is, the *y*-axes in Fig. 4 are adjusted for the different curves to have the same scale. The lower and upper data in each figure show the ESR spectrum of each MIS diode before and after *V*_G_ application, respectively. The middle data shows the ESR spectrum of each MIS diode under *V*_G_ application. When *V*_G_ = 0 V before *V*_G_ application, as shown by the lower data in each figure, no ESR signal was observed except for Fig. 4d, which means no doping of molecules due to extrinsic effects such as oxygen etc. before *V*_G_ application. In contrast, a clear signal was observed with *V*_G_; an example of the signal is shown by the middle data in each figure. The ESR parameters, *g-*factor and peak-to-peak ESR linewidth Δ*H*_pp_, of each observed ESR spectrum have been measured and are summarized in Table 1. All spectra in Fig. 4 show an almost single resonance line. The observed signal in each figure has the same *g-*factor and Δ*H*_pp_, and only the intensity depends on *V*_G_. Here, we discuss the features of each spectrum using *g-*factor and Δ*H*_pp_, and do not discuss the dependence of the intensity or number of spins in the devices on *V*_G_ because the information of the *g-*factor and Δ*H*_pp_ is enough to identify the charge species. Figure 4a,b show the hole accumulation in ADN and TBD, respectively, and Fig. 4c,d show the electron accumulation in ADN and Alq_3_, respectively. These charges are electrically accumulated at a few voltages, which confirms the high-charge-accumulation capability by the ion-gel^31^. It should be noted that the ESR signals due to the electrically accumulated holes and electrons in ADN and holes in TBD have been clearly observed for the first time by using the high-capacitance ion-gel gated devices. The ESR studies with MIS diodes with conventional solid insulators could not show such clearly electrically-induced ESR signals because of low-capacitance solid insulators compared to the high-capacitance ion-gel insulator.

**Table 1:** | Experimentally obtained ESR parameters from operando ESR spectra.

Molecular state	*g-*factor	Δ*H*_pp_ (mT)
ADN radical cation	2.0026 ± 0.0001	0.12 ± 0.01
TBD radical cation	2.0028 ± 0.0001	0.06 ± 0.01
ADN radical anion	2.0026 ± 0.0002	0.69 ± 0.01
Alq_3_ radical anion	2.0036 ± 0.0001	0.93 ± 0.01

For the case in Fig. 4d, one can notice a weak signal with *g* = 2.0028 ± 0.0002 at *V*_G_ = 0 V before *V*_G_ application. The origin of the signal has been ascribed to Alq_3_ radical anions formed by electron doping due to the reaction at the interfaces of Alq_3_/LiF/Al layer: 3LiF + Al + 3Alq_3_ → AlF_3_ + 3Li^+^Alq_3_^−^^32^. In this case, Li atom is a light element with small spin-orbital interaction and has no large contribution to the *g*-factor shift, which results in a good agreement with the theoretical calculation for Alq_3_ radical anions shown in Table 2.

**Table 2:** Calculated principal values of the *g*-tensor (*g*_1_, *g*_2_, *g*_3_) for ADN, TBD, and Alq_3_ from the DFT calculation.

Calculated molecular state	*g*_1_	*g*_2_	*g*_3_	*g*_ave_
ADN radical cation	2.00238	2.00241	2.00315	2.00265
TBD radical cation	2.00246	2.00289	2.00317	2.00284
ADN radical anion	2.00153	2.00267	2.00300	2.00240
Alq_3_ radical anion	2.00235	2.00301	2.00319	2.00285
Alq_3_ radical anion coordinated with EMIM	2.00177	2.00274	2.00511	2.00321

For ADN, radical cationic and anionic states are calculated. For TBD, a radical cationic state is calculated. For Alq_3_, a radical anionic state is calculated. An average of the principal values *g*_avg_ was calculated as ${g}_{\text{ave}}=\sqrt{{g}_{1}^{2}\langle {l}^{2}\rangle +{g}_{2}^{2}\langle {m}^{2}\rangle +{g}_{3}^{2}\langle {n}^{2}\rangle }$ using $l = \sin \theta \cos \phi$ ,$m = \sin \theta \sin \phi$ , and $n = \cos \theta$ , where <  > represents spatial average. Here random orientation of molecules was assumed to calculate the *g*_ave_.

We here present the *V*_G_ response of the ESR signal. As shown by the upper data in Fig. 4a,b, when the *V*_G_ was returned to 0 V, the ESR signals due to electrically accumulated holes in ADN and TBD disappeared completely. These data confirm that the hole accumulation shows complete reversibility upon the *V*_G_ application. In contrast, for the electron accumulation in ADN and Alq_3_, a considerably large or small ESR signal remained after turning off the *V*_G_, as shown by the upper data in Fig. 4c,d, respectively. The residual ESR signals will be discussed later in detail.

To examine the molecular orientation for the charge-accumulation sites in the organic materials, we measured the anisotropy of the ESR signals of all MIS diodes with respect to the direction of the external magnetic field *H* to the substrate plane. Although the *H* direction was varied from parallel to perpendicular to the substrate plane at the 15° intervals, we observed no ESR anisotropy within the error bars of the *g-*factor (0.0001–0.0002) and Δ*H*_pp_ (0.01 mT) (see Table 1). Therefore, this anisotropy demonstrates that the molecules where charges electrically accumulate are amorphous, not oriented, in the thin-films. Such amorphous nature has been often observed for OLED materials^32^.

### DFT analysis of spin-states of electrically accumulated charges

**Figure 5:**
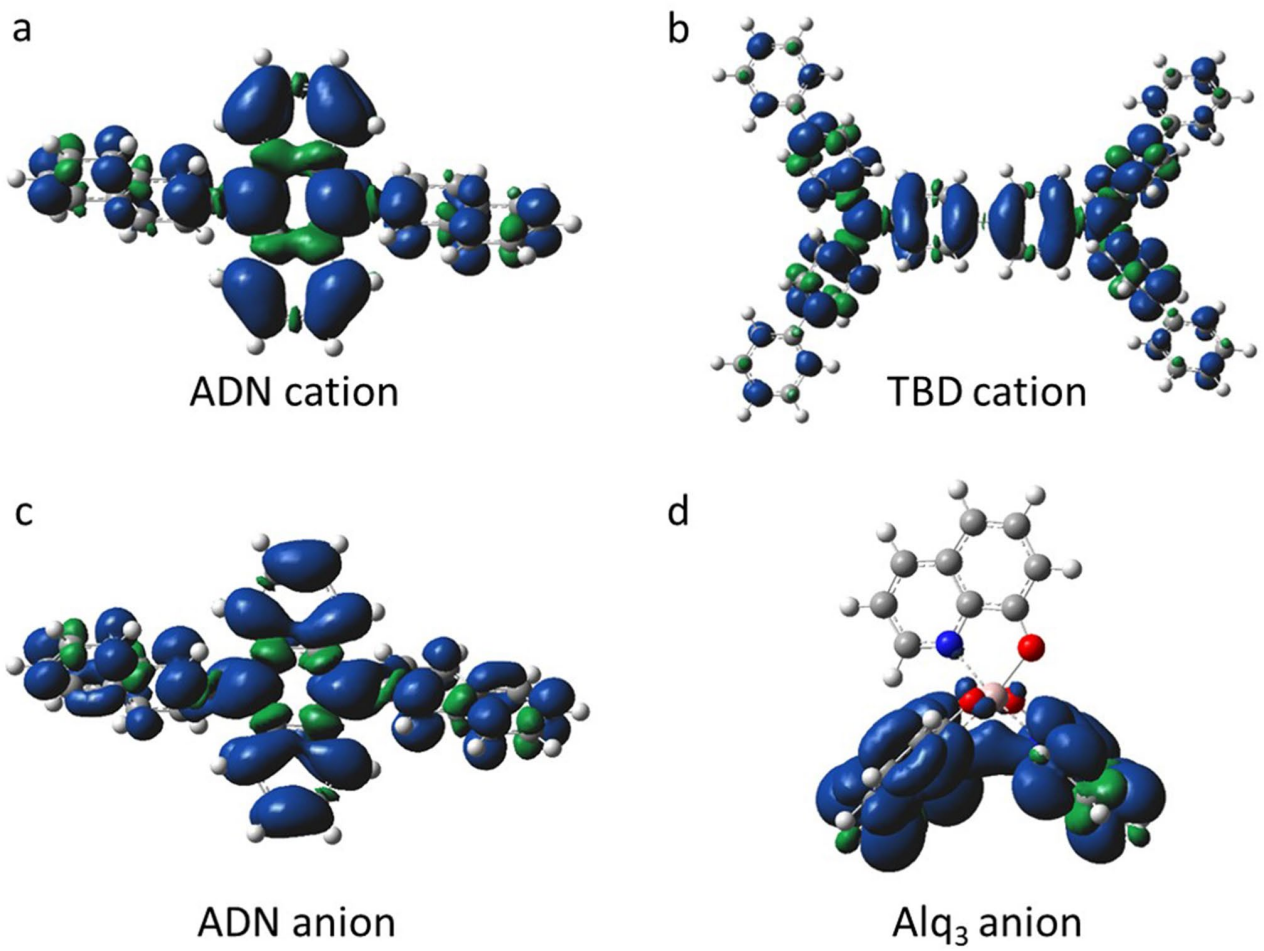
Spin-density distribution of doped organic materials obtained from the DFT calculation. (**a**) ADN cation. (**b**) TBD cation. (**c**) ADN anion. (**d**) Alq_3_ anion.

To analyze the *g*-factors obtained from the ESR spectra, DFT calculation was performed. We used the B3LYP functional and the 6-31G+(d,p) basis set for the DFT calculation with Gaussian 09^38^. For the DFT calculation, the cation state of the TBD monomer, the cation and anion states of the ADN monomer, and the anion state of the Alq_3_ monomer were calculated under structural optimization conditions. The obtained principle values (*g*_1_, *g*_2_, *g*_3_) of the *g*-tensors are summarized in Table 2, and the spin-density distributions of these states are shown in Fig. 5. As shown in Fig. 5, the spin-density spatially spreads over the molecules, except for the Alq_3_ anion. For the Alq_3_ anion, the present results of the principle values and spin-density distribution that are obtained with the B3LYP/6-31G+(d,p) level are almost consistent with those obtained by the previous work with the B3LYP/6-31G(d) level^39^.

The organic molecules with charge accumulation have amorphous nature for molecular orientation, as mentioned above. Thus, to compare the calculated *g*-factors (Table 2) with the experimental values (Table 1), the average value of the principle values, *g*_ave_, was calculated. For the ADN and TBD radical cations, the calculated values of *g*_ave_ = 2.00265 and *g*_ave_ = 2.00284 are well consistent with the experimental value of *g* = 2.0026 ± 0.0001 and *g* = 2.0028 ± 0.0001 within the error bars, respectively. For the ADN radical anion, the calculated value (*g*_ave_ = 2.0024) is almost consistent with the experimental value (*g* = 2.0026 ± 0.0002) within the error bars. Since the ADN molecules have no heteroatoms such as N and O, charge-conjugation symmetry (CCS) for electrons and holes is maintained^40,41^. This CCS is reflected in the spin-density distribution of the ADN radical cation and anion which is similar with each other as shown in Fig. 5a,c. For Alq_3_, the calculated value (*g*_ave_ = 2.00285) is considerably smaller than the experimental value (*g* = 2.0036 ± 0.0001). The reason for the larger experimental value may be due to an effect of spin–orbital interaction in a positive EMIM ion in the ion-gel and the Alq_3_ anion^42^. That is, an electron on Alq_3_ spreads over the positively charged EMIM, which may increase the *g*-factor by the enhanced spin–orbital interaction. This effect has been supported by the DFT calculation for Alq_3_ coordinated with EMIM (Table 2; Figure S1 in Supplementary Information). In this case, the *g*_ave_ is calculated to be 2.00321 (Table 2), which is larger than that of Alq_3_ anion (*g*_ave_ = 2.00285) and is closer to that of experimental value (*g* = 2.0036). Figure S1 in Supplementary Information shows the calculated spin-density distribution, which shows a different distribution compared to the case without EMIM coordination (Fig. 5d).

The coordination effect with EMIM seems to strongly occur for the case of Alq_3_ anions, as mentioned above. However, such effect seems not to strongly occur for the case of ADN anions because the calculated value (*g*_ave_ = 2.0024) by DFT is almost consistent with the experimental value (*g* = 2.0026), as mentioned above. The reason for the different coordination effect may be due to the different molecular structures between Alq_3_ and ADN. That is, the Alq_3_ molecule has oxygen atoms with negative electrostatic property, which may tend to strongly coordinate with EMIM cations. In contrast, the ADN molecule has no oxygen atoms with negative electrostatic property, which may result in no strong coordination with EMIM cations. Thus, the results for ADN anions obtained with the MIS diodes will be applicable to the electronic states in the blue OLEDs because of no strong coordination effect between ADN anions and EMIM cations in the MIS diodes.

### Charge-trappings in organic layers

Next, we discuss the charge-trappings in organic layers. We have studied the charge injection from the contact electrode to the organic layer by measuring the time evolution of the ESR signal from the beginning of the *V*_G_ application to the device. It has been known that the ESR lineshape varies depending on whether the charge with a spin is static or mobile^28^. This is because hyperfine interactions occur between charges’ spins and nuclear spins in organic molecules, which determines the linewidth of the ESR signals^39^. When the charge with a spin is in motion, the hyperfine interactions are averaged, and the motional narrowing of the ESR linewidth has been reported^43^.

**Figure 6:**
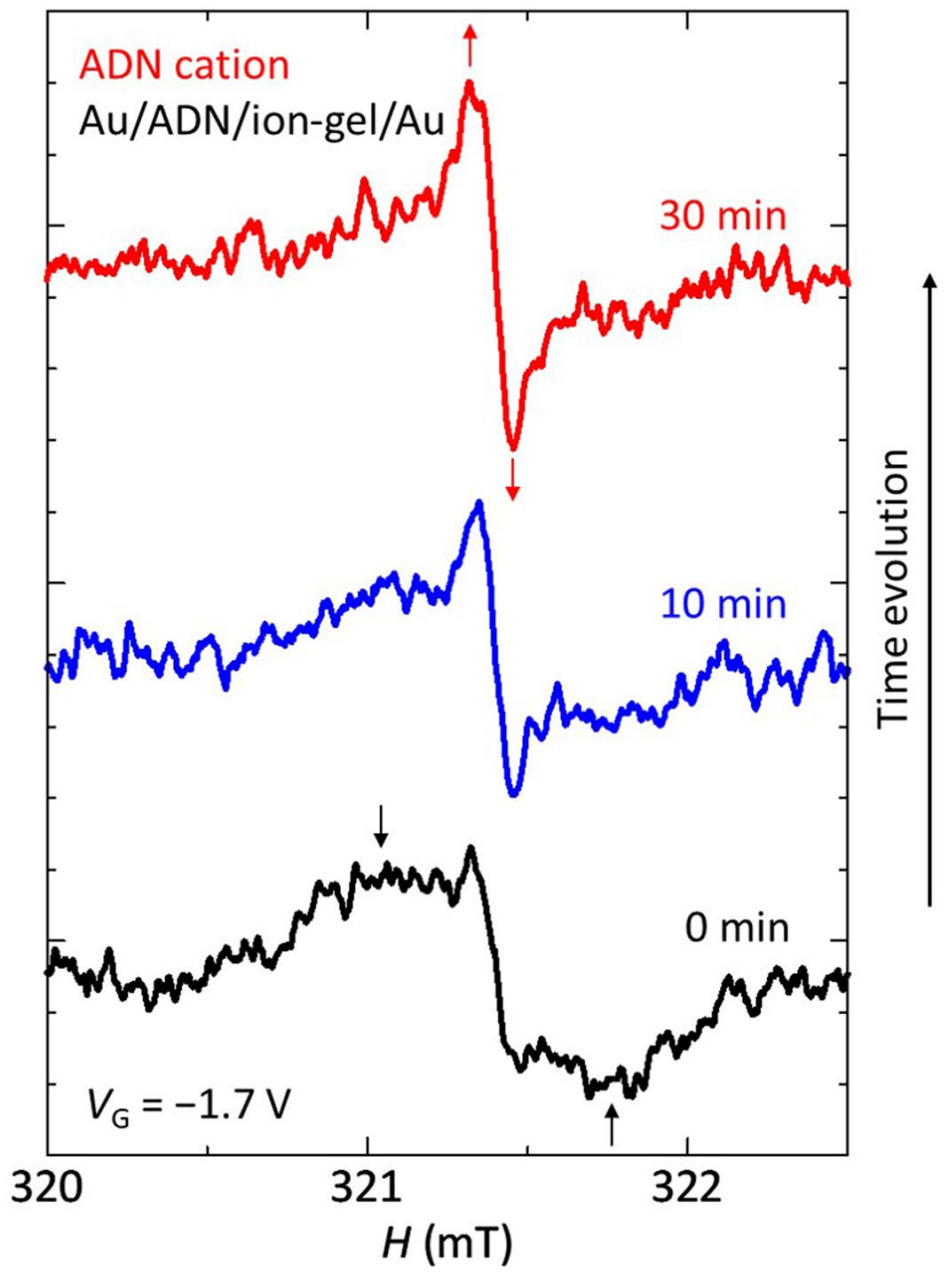
Time evolution of the ESR spectrum of the ADN MIS diode during device operation. These data show the ESR spectrum at *V*_G_ =  − 1.7 V just after *V*_G_ application (lower data), after 10 min *V*_G_ application (middle data), and after 30 min *V*_G_ application (upper data), respectively.

Figure 6 shows an example of the time evolution of the ESR spectrum of the ADN MIS diode, showing the charge-accumulation process. When the |*V*_G_| was increased from 0 V at an interval of 0.1 V, the charge injection occurred at a threshold voltage of − 1.7 V, where holes were injected from the contact Au electrode, and corresponding ESR signals were observed. Just after applying *V*_G_ =  − 1.7 V, the signal component with a broad linewidth was immediately observed in addition to the signal component with a narrow linewidth; the signal with the broad linewidth is indicated by black arrows for the lower data (0 min) in Fig. 6. After that, when the *V*_G_ was fixed at − 1.7 V, the signal component with the narrow linewidth became larger, as shown by the middle data (10 min). This narrow component was clearly enhanced as indicated by red arrows for the upper data (30 min) in Fig. 6. This charge-accumulation process can be explained as follows. At the threshold voltage, holes are firstly injected and trapped at the trapping levels in the ADN layer. In this case, the charge motion is hindered, so that the motional narrowing does not occur and the linewidth remains broad. That is, the trapped and static charges with spins directly feel hyperfine interactions from nuclear spins in the ADN layer, which makes the linewidth surprisingly broader. After that, some charges are de-trapped from the trapping levels at a certain rate and move in the organic layer, which narrows the linewidth due to the average of the hyperfine interactions by the motion of charges with spins, as mentioned above. The time constant of the complete de-trapping seems to be longer than several minutes, as shown in Fig. 6. This result demonstrates that the charges are trapped in the ADN layer just after the charge injection and that the subsequent charge motion is directly observed from a microscopic viewpoint. The long time constant of the complete de-trapping may be due to the charge-trapping sites with deep trapping levels at the interface between the metal electrode and organic molecules. That is, the charges are firstly injected by the electrostatic attraction from ions in the ion-gel and are trapped in the deep trapping levels at the metal/organic interface. However, the electrostatic potential for the trapped charges from ions in the ion-gel may be minimum at another interface between organic molecules and the ion-gel. Thus, the trapped charges are de-trapped from the deep trapping levels beyond the high potential barrier due to the deep trapping levels, which may take a long time, and then move in the organic layer, as mentioned above. Similar result has been obtained for the MIS diode for TBD cations. Other MIS diodes for ADN or Alq_3_ anions have not shown such a clear change of the ESR spectrum shown in Fig. 6 because these ESR spectra have much broader linewidth, which causes lower peak-to-peak ESR intensity, compared to those of the MIS diodes for ADN or TBD cations (Fig. 4), as explained later.

For the charge-trapping sites, one can consider several possibilities, such as in bulk organic molecules, at the interface between metal electrode and organic molecules, and at the interface between organic molecules and ion-gel. To further study the charge-trapping sites, we have performed the ESR studies of a hole only device (HOD) of ITO (150 nm)/TBD (100 nm)/ADN (60 nm)/Au (50 nm), and an electron only device (EOD) of ITO (150 nm)/ADN (60 nm)/Alq_3_ (4 nm)/LiF (0.5 nm)/Al (100 nm). However, we could not observe a very clear change of ESR signals for the HOD and EOD after 1 h device operation compared to the cases with the MIS diodes. This result can be attributed to the smaller number of long-lived accumulated charges in the HOD and EOD than that in the MIS diodes with high-capacitance ion-gel; the flow of electric current shortens the lifetime of charges in the HOD and EOD than < 10 μs. Thus, we have not definitely identified further detailed charge-trapping sites such as in the bulk material and/or at the interface. However, the above discussion for Fig. 6 may indicate the charge-trapping sites at the metal/organic interface.

The previous study has reported the carrier drift mobilities in ADN films with 5–7 μm thickness by using optical time-of-flight (TOF) technique, where both electron and hole mobilities of ADN are the same in the range of 1–4 × 10^−7^ cm^2^ V^−1^ s^−1^ (electric field 0.5–0.8 MV/cm)^44^. In the case with the TOF method, the drift mobility is derived from the averaged running velocity over whole film with a rather thick film of 5–7 μm thickness. The ADN films have been reported to be amorphous^44^. Thus, the photogenerated charges encounter many grain boundaries when they move in the film, and the evaluated mobility may be affected by such grain boundaries, which may give the similar mobility between electrons and holes. In contrast, the above-mentioned motional narrowing of the ESR linewidth reflects the local motion of charges in organic molecules, without being affected by such grain boundaries. Thus, the narrower the ESR linewidth, the higher the local mobility in organic molecules. Figure 4a,c show that the ESR linewidth of ADN cations (Δ*H*_pp_ = 0.12 mT) is narrower than that of ADN cations (Δ*H*_pp_ = 0.69 mT). This result indicates that the local mobility of ADN cations is higher than that of ADN anions from a microscopic viewpoint.

### Charge-trappings in organic multilayers

**Figure 7:**
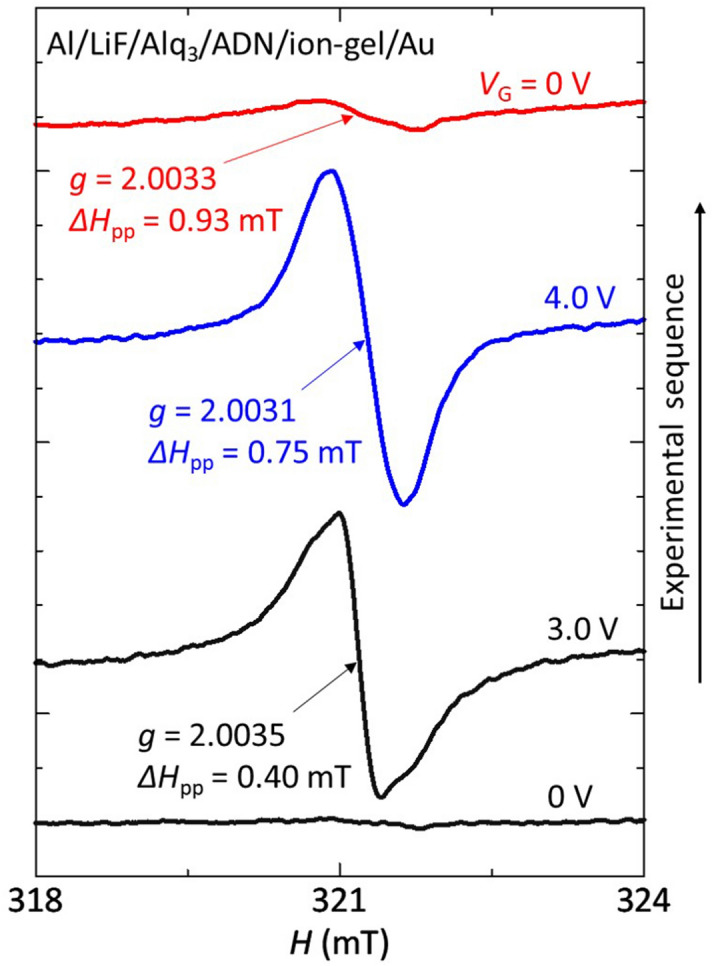
*V*_G_ dependence of the ESR spectrum of an organic multilayer MIS diode. These data show the ESR spectrum before *V*_G_ application (*V*_G_ = 0 V, lower data), at *V*_G_ = 3.0 V (second lower data), at *V*_G_ = 4.0 V (second upper data), and after *V*_G_ application (*V*_G_ = 0 V, upper data), respectively.

Finally, we discuss the charge-trappings in an organic multilayer MIS diode (Al/LiF/Alq_3_/ADN/ion-gel/Au, Fig. 3d). This organic multilayer is the same as that in the blue OLED of ITO/TBD/ADN/Alq_3_/LiF/Al (Fig. 1c). Thus, clarifying the charge states in the organic multilayer will be useful for understanding the behavior of the OLED further. Figure 7 shows the *V*_G_ dependence of the ESR spectrum of the organic multilayer MIS diode, which shows the electron accumulation and discharge. When *V*_G_ = 0, no ESR signal was observed (the lower data), except for the signal due to Li^+^Alq_3_^−^ as discussed for the lower data in Fig. 4d. As the *V*_G_ increased, the charge injection occurred at the threshold voltage of *V*_G_ = 3.0 V and an ESR signal with *g* = 2.0035 ± 0.0001 was clearly observed (the second lower data). This threshold voltage is almost the same as that of the Alq_3_ MIS diode (Fig. 4d). If looking carefully at the data of *V*_G_ = 3.0 V, one can notices a weak structure in the spectrum. This result indicates the overlapping of two ESR signals of Alq_3_ anions and ADN anions because the observed *g*-factor (*g* = 2.0035) is slightly smaller than that of Alq_3_ anions (*g* = 2.0036, Fig. 4d) but is larger than that of ADN anions (*g* = 2.0026, Fig. 4c). As the *V*_G_ further increased to 4.0 V, the signal intensity increased and the *g*-factor slightly decreased to *g* = 2.0031 ± 0.0001 (the second upper data in Fig. 7). This *g*-factor decrease demonstrates that the ESR intensity of ADN anions becomes larger compared to that at *V*_G_ = 3.0 V because the *g*-factor of ADN anions (*g* = 2.0026) is smaller than that of Alq_3_ anions (*g* = 2.0036), which indicates that ADN anions are largely formed at high *V*_G_ of 4.0 V. After that, when we stopped applying the *V*_G_, the signal intensity decreased and an ESR signal with *g* = 2.0033 ± 0.0001 was still observed (the upper data in Fig. 7). This result indicates that ADN anions in addition to Alq_3_ anions remain in the organic layers even after turning off the *V*_G_ because the observed *g*-factor is smaller than that of Alq_3_ anions (*g* = 2.0036, Fig. 4d).

For Alq_3_-based OLEDs, it has been reported that the formation of Alq_3_ anions in the device does not contribute to the luminance degradation while Alq_3_ cations degrade the device performance^9,45^. If the formation of Alq_3_ anions in the blue OLED does not contribute to the luminance degradation in the same way, ADN anions in the device will cause the luminance degradation. The ESR studies of the ADN MIS diodes have shown that the accumulated electrons remained (Fig. 4c) while no holes remained in ADN after turning off the *V*_G_ (Fig. 4a). Therefore, for the ADN OLED, the formation of radical anions will be an important degradation factor compared to the case with radical cations because of the reasons such as the molecular degradation and carrier-balance loss in the device, resulting in the degradation of the devices characteristics. Obtaining the proof of the molecular degradation of ADN such as the change of the molecular structure in the device is an interesting issue, which may be performed by the detailed analysis of the ESR spectrum because the values of the ESR linewidth of the ADN anion signal are different as shown in Fig. 4c. That is, the Δ*H*_pp_ is 0.69 mT at *V*_G_ = 4.2 V (the middle data in Fig. 4c) while it is 1.20 mT at 0 V (the upper data in Fig. 4c after *V*_G_ application), which may indicate a change of the molecular structure of ADN. This difference may be explained by performing the detailed DFT analysis with assumed degraded molecular structures, which is in progress and will be reported in a separate paper.

We comment on an ESR study of hole injection and discharge in another organic multilayer MIS diode (Ni/Au/TBD/ADN/ion-gel/Au, Fig. 3c). In this case, we have mainly observed the ESR signal of TBD cations; almost no clear change of the *g*-factor was observed when the |*V*_G_| increased. This result may be explained by the larger peak-to-peak ESR intensity *I*_pp_ of the ESR spectrum of TBD cations compared to that of ADN cations considering the noise levels as shown in Fig. 4a,b, which can be ascribed to the narrower Δ*H*_pp_ of TBD cations (0.06 mT) compared to that of ADN cations (0.12 mT). A number of spins evaluated from an ESR spectrum is proportional to *I*_pp_ × (Δ*H*_pp_)^2^, and thus the narrower the Δ*H*_pp_, the larger the *I*_pp_. After turning off the *V*_G_, no ESR signal was observed, which is consistent with those for the ADN or TBD MIS diodes (the upper data in Fig. 4a,b).

To study the charge-trappings in blue OLEDs, we have performed an ESR study of the blue OLEDs of ITO (150 nm)/TBD (100 nm)/ADN (60 nm)/ Alq_3_ (4 nm)/LiF (0.5 nm)/Al (100 nm) (Fig. 1c). However, we could not observe a very clear change in the ESR signal of the blue OLED after 1 h device operation compared to the cases with the MIS diodes. As mentioned in the studies of the HODs and EOD, this result can be attributed to the smaller number of long-lived accumulated charges in the blue OLEDs than that in the MIS diodes with the high-capacitance ion-gel; the flow of electric current shortens the lifetime of charges in the OLEDs.

## Conclusion

We have performed the operando ESR study of the organic single- or multi-layer MIS diodes, HOD, EOD, and blue OLEDs to elucidate the spin-states in the organic semiconductor materials used for the blue OLEDs. For the organic single-layer MIS diodes, no ESR signal was observed at *V*_G_ = 0 V before *V*_G_ application except for the Alq_3_ MIS diode, while clear ESR signal was obtained in each device by applying the *V*_G_. The observed *g*-factors are well reproduced by the DFT calculation. We demonstrate the charge-trappings in the organic layers from a microscopic viewpoint. The trapping levels of electrons has been found to be deeper than those of holes; the electron accumulation in ADN is confirmed for the organic multilayer MIS diodes. In contrast to the case of the green light-emitting material Alq_3_, long-lived radical anions in the blue light-emitting material ADN will contribute the degradation of the molecules and devices, and thus blue ADN OLEDs with higher efficiency and longer lifetime will be developed by decreasing or eliminating the ADN electron accumulation. Decreasing or eliminating the ADN electron accumulation is an interesting topic, which is currently studied by optimizing of molecules and device structures and will be reported in a separate paper.

## Methods

### Fabrication of organic devices

The MIS devices were fabricated using two types of nonmagnetic substrates; one was a polyethylene terephthalate (PET) film with dimensions of 30 mm × 3 mm × 100 μm (Mitsubishi Polyester Film, Inc.), and the other was a quartz substrate with dimensions of 30 mm × 3 mm × 1 mm (IIYAMA PRECISION GLASS Co, Ltd.). Gate electrodes of Ni/Au (3/47 nm) were vapor-deposited on the PET substrate using an ULVAC VPC-260F vacuum evaporation system. Ion-gel solutions consisted of an ionic liquid, 1-ethyl-3-methylimidazolium bis(trifluoromethylsulfonyl)imide ([EMIM][TFSI]) (52.2 wt%) (Ionic Liquids Technologies, Inc.), a gelator ABA-type triblock copolymer poly(styrene-*b*-methylmethacylate-*b*-styrene) (PS-PMMA-PS) (4.3 wt%) (Polymer Source, Inc.), and a solvent ethyl acetate (43.5 wt%) (Wako Pure Chemical Industries, Ltd.); the mixture was stirred for over one and half day, drop-casted on the gate electrode and then thermally annealed at 70 °C under vacuum for over one and half day. The contact electrodes of Ni/Au (3 nm/47 nm) or Al/LiF (100 nm/0.5 nm) for the hole or electron accumulation were fabricated with the vapor-deposition system on the quartz substrate, respectively. The TBD, ADN, and Alq_3_ organic layers with a film thickness of 100 nm, 60 nm, and 60 nm were vapor-deposited on the contact electrodes with the vacuum evaporation system under 5 × 10^−4^ Pa, respectively. Finally, the PET substrate was placed on the quartz substrate, completing the device fabrication. The fabricated device was sealed into an ESR sample tube after wiring with Ag paste in a nitrogen-filled glove box (O_2_ ≤ 0.5 ppm, H_2_O ≤ 0.5 ppm). The parts of the above-mentioned fabrication method have been described in the previous works^31,35,46–49^.

### Device characterization

The ESR measurements were performed with a JEOL RESONANCE JES-FA200 ESR spectrometer (X-band 9.5 GHz) and a Keithley 2612A source meter. The ESR signals were measured as a function of *V*_G_ by averaging the ESR spectrum over typically 10–15 min. The *g*-factor and linewidth of the ESR signals were calibrated using a standard Mn^2+^ marker sample. The calibration of the *g*-factor was performed with a software program of the JEOL RESONANCE ESR system, considering high second-order correction to the effective resonance field. Its correctness was also confirmed with 2,2-diphenyl-1-picrylhydrazyl (DPPH) as another standard sample. The parts of the above-mentioned characterization method have been described in the previous works^30–35,46–49^.

## Supplementary information

Supplementary Figure

## Data Availability

The authors declare that the data supporting the findings of this study are available within the paper and its supplementary information file.
