# Supplementary material for: Operando direct observation of spin-states and charge-trappings of blue light-emitting-diode materials in thin-film devices
Source: Sci Rep. 2020 Nov 2;10:18800. doi: 10.1038/s41598-020-75668-4 (PMC7606584; doi:10.1038/s41598-020-75668-4)
Supplement: Supplementary file 1 — Supplementary Figure [file 41598_2020_75668_MOESM1_ESM.pdf]

Supplementary Information for

Operando direct observation of spin-states and charge-trappings of blue light-emitting-diode materials in thin-film devices

Fumiya Osawa and Kazuhiro Marumoto*

*Correspondence: marumoto@ims.tsukuba.ac.jp

S1. DFT calculation of Alq_3 anion coordinated with EMIM

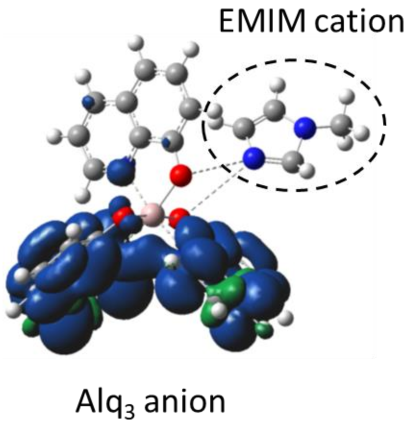

Figure S1 | Spin-density distribution of Alq_3 anion coordinated with EMIM component obtained from the DFT calculation.
